# Using early childhood infections to predict late childhood antibiotic consumption: a prospective cohort study

**DOI:** 10.3399/bjgpopen20X101085

**Published:** 2020-10-21

**Authors:** Kristian Gjessing, Johnny Ludvigsson, Åshild Olsen Faresjö, Tomas Faresjö

**Affiliations:** 1 Department of Medicine and Health, Medical Faculty, Linköping University, Linköping, Sweden; 2 Crown Princess Victoria Children’s Hospital, Linköping University, Linköping, Sweden; 3 Division of Paediatrics, Department of Clinical and Experimental Medicine, Linköping University, Linköping, Sweden

**Keywords:** Anti-Bacterial Agents, Prescriptions, Child, Prospective Studies, Socioeconomic Factors, Primary Health Care

## Abstract

**Background:**

In the Swedish welfare system, the prescription and price of antibiotics is regulated. Even so, socioeconomic circumstances might affect the consumption of antibiotics for children.

**Aim:**

This study aimed to investigate if socioeconomic differences in antibiotic prescriptions could be found for children aged 2–14 years, and to find predictors of antibiotic consumption in children, especially if morbidity or socioeconomic status in childhood may function as predictors.

**Design & setting:**

Participants were from All Babies In Southeast Sweden (ABIS), a prospectively followed birth cohort (*N* = 17 055), born 1997-1999. Pharmaceutical data for a 10-year period, from 2005–2014 were used (the cohort were aged from 5–7, up to 14–16 years). Participation at the 5-year follow-up was 7443 children. All prescriptions from inpatient, outpatient, and primary care were included. National registries and parent reports were used to define socioeconomic data for all participants. Most children’s infections were treated in primary healthcare centres.

**Method:**

Parents of included children completed questionnaires about child morbidity at birth and at intervals up to 12 years. Their answers, combined with public records and national registries, were entered into the ABIS database and analysed. The primary outcome measure was the number of antibiotic prescriptions for each participant during a follow-up period between 2005–2014.

**Results:**

The most important predictor for antibiotic prescription in later childhood was parent-reported number of antibiotic-treated infections at age 2–5 years (odds ratio (OR) range 1.21 to 2.23, depending on income quintile; *P*<0.001). In the multivariate analysis, lower income and lower paternal education level were also significantly related to higher antibiotic prescription.

**Conclusion:**

Parent-reported antibiotic-treated infection at age 2–5 years predicted antibiotic consumption in later childhood. Swedish doctors are supposed to treat all patients individually and to follow official guidelines regarding antibiotics, to avoid antibiotics resistance. As socioeconomic factors are found to play a role, awareness is important to get unbiased treatment of all children.

## How this fits in

Previous research has shown that antibiotic prescription rates vary with geography, socioeconomy, patient morbidity, and local medical culture. Also known is that younger children have more infections and receive more antibiotic prescriptions than older children. This study adds that the number of antibiotics-treated infections (as reported by parents) in early childhood seems to predict antibiotic consumption in later childhood. Even in an egalitarian country with strict guidelines for antibiotic use, socioeconomic and psychosocial factors may influence prescription.

## Introduction

Nordic healthcare systems are intended to provide equal access to care, based on individual needs, regardless of the patient’s economic resources.^1^ However, present illness is not the only factor that influences the demand for care. Healthcare utilisation is also determined by the healthcare system structure, access to health care and pharmaceuticals, and the health insurance system. Individual sociocultural and socioeconomic factors might also affect demand and use of health care. One example of this is antibiotics prescription to children, which might be influenced by other factors than just the child's present infection.^2^ The majority of children with infections are treated in primary care.^3^

Socioeconomic influences on antibiotics prescription rates have been described, with contrasting results. Children with lower socioeconomic status tend to be more vulnerable to respiratory infections, possibly needing more antibiotics and receiving more prescriptions.^4–7^ Parental smoking, closely linked to socioeconomic factors, has been shown to increase the risk of acute otitis media in children.^8^ Melander *et al* ^9^ showed how adjacent geographical areas could have diverging relationships between children’s antibiotic use and the municipal level of adult education. Socioeconomic differences in day-care arrangements have been associated with use of medical care and antibiotics in Swedish preschool children.^10^ Danish children were shown to use more antibiotics as parents’ educational level decreased.^11^ The family’s economic status may directly affect the willingness to buy prescribed drugs.^12^

The expectations by patients or their family could influence the physician’s decision to prescribe antibiotics or not,^13–16^ and this 'family medical culture' may become more evident when the patient is a child, as children are more prone to infections than adults.^3^ Even organisational factors, such as whether the healthcare centre has short-term/locum doctors or regularly employed doctors, may impact the rates of antibiotic prescribing.^17,18^

The discussion about the use of antibiotics is ongoing. National evidence-based guidelines in Sweden recommend strict criteria for the use of antibiotics.^19^ Antibiotics usage is generally lower in Sweden than in many other countries,^20^ but other moderating factors might still impact. Hedin *et al* ^2^ found regional cultural differences in antibiotic prescription patterns. Nilsson and Laurell^7^ found higher antibiotic prescription rates in high-income residential areas of a large Swedish city, while Pini *et al* recently found higher morbidity rates in less advantaged groups in Sweden.^21^ Given this background, this study wanted to investigate predictive factors for antibiotic consumption in a cohort of Swedish children, as these results might show a need for new strategies to overcome inequalities.

## Method

### Participants

**Figure 1.**
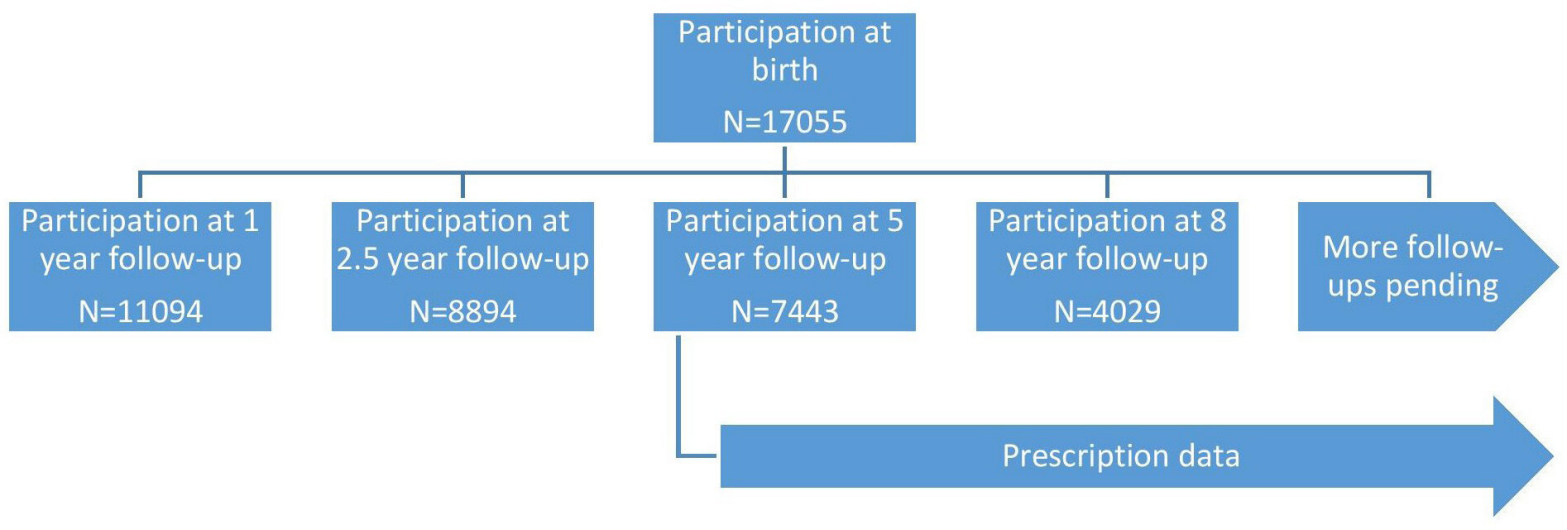
Number of participants and follow-ups in the ABIS study. Prescription data are from routine health records and are therefore complete

ABIS is a prospective birth cohort study consisting of children born in the southeast region of Sweden, between 1 October 1997 and 1 October 1999. The parents of 17 055 children (78.6% of the eligible cohort) gave informed consent to participate. The ABIS database contains a unique personal ID number, thus every individual can be followed through all healthcare encounters, including diagnoses and prescriptions. National data were linked to this study's database using the same personal ID number. Since 2001, the ABIS database has been used in more than 100 published studies, according to its website.^22^ The longitudinal study design, with number of participants at each follow-up, is shown in Figure 1. A general prospective study protocol directs the different ABIS projects. Some general sociodemographic data about the cohort are presented in Table 1.

**Table 1.** Sociodemographic description of the participating children at 5-year follow-up (*N* = 7443)

**Sociodemographic data**	**Participating children at 5-year follow-up**
	***n***	**%**
**Sex**
Male	3885	52.2
Female	3558	47.8
**Birth weight <2.500 grams**	211	2.8
**Small for gestational age**	147	2.0
**Born in or before week 36**	153	2.1
**Mother smoking during pregnancy**	586	7.9
**Mother’s education level^a^**
9 years / primary school	465	6.4
12 years / high school	4293	59.0
University degree	2518	34.6
**Father’s education level^b^**
9 years / primary school	915	12.7
12 years / high school	4484	62.3
University degree	1804	25.0
**Mother’s place of birth^c^**
Born in Sweden	7132	98.0
Born outside of Sweden	148	2.0
**Parental civil status^c^**
Married	3279	45.0
Cohabitant	3913	53.8
Single parent	88	1.2
**Received antibiotics prescription during 2005** **–** **2014**	6231	83.7
**Median age of mothers at childbirth, years (range)**	29 (16 to 45)	
**Median birth weight, grams**	3595	

^a^ *N* = 7276. ^b^ *N* = 7203. ^c^ *N* = 7280.

The Swedish Prescribed Drug Register (SPDR) gathers data through automatic reporting from all Swedish pharmacies, every time a prescription is filled (the prescribed drug is delivered from the pharmacy). Data of all prescriptions made to the children in this cohort were linked to this study's database. In this study, pharmaceutical data for a 10-year period, from 2005–2014 were used (the cohort were aged from 5–7, up to 14–16 years).

The registry for the following Anatomical Therapeutical Chemical (ATC) classification^23^ groups was searched: penicillins with extended spectrum (J01CA); beta-lactamase sensitive penicillins (J01CE); beta-lactamase resistant penicillins (J01CF); amoxicillin and beta-lactamase inhibitor (J01CR02); ceftibuten (J01DD14); erythromycin (J01FA01); azithromycin (J01FA10); clindamycin (J01FF01); and nitrofurantoin (J01XE01). This list of prescription drugs represents approximately 86% of all prescriptions in this study's material, and covers the most common and recommended oral antibiotics for children in Swedish primary health care. In total, 10 414 children (65% of this ABIS cohort) had received and filled an antibiotic prescription during 2005–2014. For this article, it was assumed that filled prescriptions equals consumption. The number of antibiotic prescriptions per child varied between none and 78 prescriptions.

**Figure 2.**
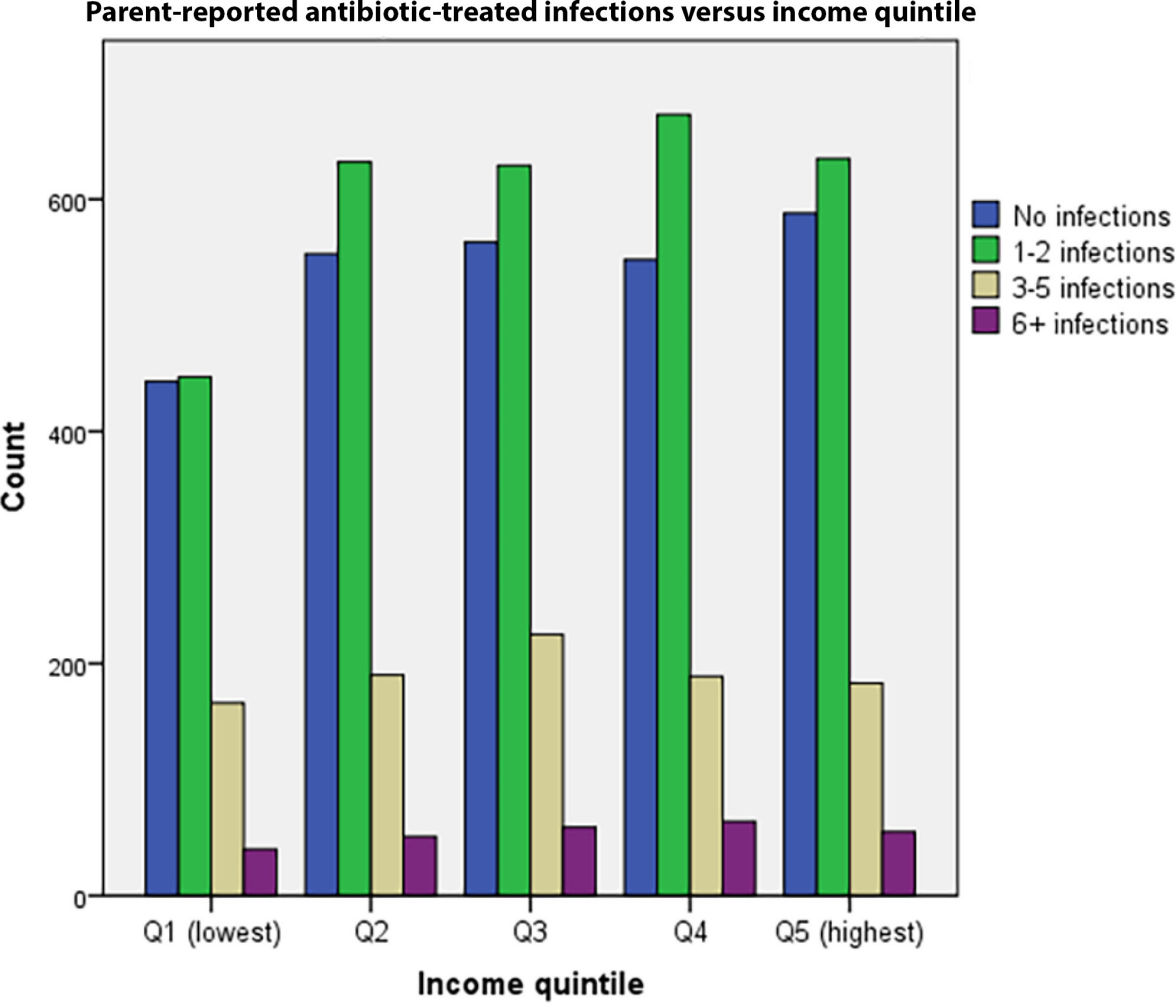
The distribution of parent-reported infectious morbidity for children aged 2–5 years shows that most children have had a low number of infections. The number of children with no, or 1–2 infections is lower for income quintile one than for higher income quintiles

As a proxy for infectious morbidity, parent-reported illness of the child were used: at 5-years-old, parents were asked to report how many infections that required antibiotics the child had had during the last 3 years. Alternatives were 0, 1–2, 3–5, and ≥6 infections (Figure 2).

Household income data were from Statistics Sweden for 2006, and were divided into quintiles. Parent-reported data regarding the mother’s and father’s education were classified into three levels; low (primary school, up to and including year 9), medium (high school/college, 12 years), or high (university level).

### Statistics

The dependent variable for all analyses was count of antibiotic prescriptions per child. While counts often follow a Poisson distribution, the likelihood ratio test indicated overdispersion relative to a Poisson distributed random variable, and therefore all statistical analyses are based on negative binomial regression. In Table 2, a univariate model was fitted, which treated each potential correlate of antibiotic count in isolation, and then in Table 3, a multivariable model was fitted, which considered the joint effects of several potential predictors. Potential predictors of antibiotic count were selected by backward elimination, as this takes into account the full covariance structure of the correlates. Missing data were excluded on a case-by-case basis, so only cases with complete data were included in each analysis. Correlations between all variables are shown in Supplementary Table S1. For all analyses, IBM SPSS Statistics (version 24) was used. A *P* value <0.05 was considered significant.

**Table 2.** Univariate associations between risk factors and number of antibiotic prescriptions (*N* = 6231)

Model	Odds ratio	95% CI	*P* value
**Intercept**	1.37	1.12 to 1.66	0.002
**Sex** **,** **female**	1.00	—	—
**Sex** **,** **male**	1.12	1.05 to 1.19	<0.001
**Income**			0.003
Q5 (highest quintile)	1.00	—	—
Q4	1.07	0.97 to 1.18	0.176
Q3	1.16	1.05 to 1.27	0.003
Q2	1.15	1.05 to 1.27	0.004
Q1	1.19	1.07 to 1.32	0.001
**Mother’s education level**			0.071
University	1.00	—	—
12 years / high school	1.07	1.00 to 1.14	0.060
9 years / primary school	1.14	0.99 to 1.30	0.065
**Father’s education level**			0.011
University	1.00	—	—
12 years / high school	1.11	1.03 to 1.19	0.008
9 years / primary school	1.15	1.03 to 1.28	0.010
**Parent-reported **antibiotics-treated** infections i** **n children aged** **2** **–** **5** **years**			<0.001
0	1.00	—	—
1–2	1.21	1.13 to 1.30	<0.001
3–5	1.72	1.56 to 1.89	<0.001
≥6	2.23	1.91 to 2.60	<0.001

Dependent variable: number of antibiotic prescriptions.

**Table 3.** Multivariable associations between risk factors and number of antibiotic prescriptions (*N* = 6231)

Model	Odds ratio	95% CI	*P* value
**Sex** **,** **female**	1.00	—	—
**Sex** **,** **male**	1.11	1.04 to 1.18	0.001
**Income quintile**			0.002
Q5 (highest quintile)	1.00	—	—
Q4	1.04	0.94 to 1.14	0.461
Q3	1.15	1.05 to 1.27	0.002
Q2	1.14	1.04 to 1.25	0.006
Q1	1.17	1.06 to 1.29	0.002
**Parent-reported antibiotics-treated infections in children aged 2–5 years**			<0.001
0	1.00	—	—
1–2	1.24	1.16 to 1.32	<0.001
3–5	1.72	1.57 to 1.89	<0.001
≥6	2.31	1.99 to 2.68	<0.001

Dependent variable: number of antibiotic prescriptions

## Results

**Figure 3.**
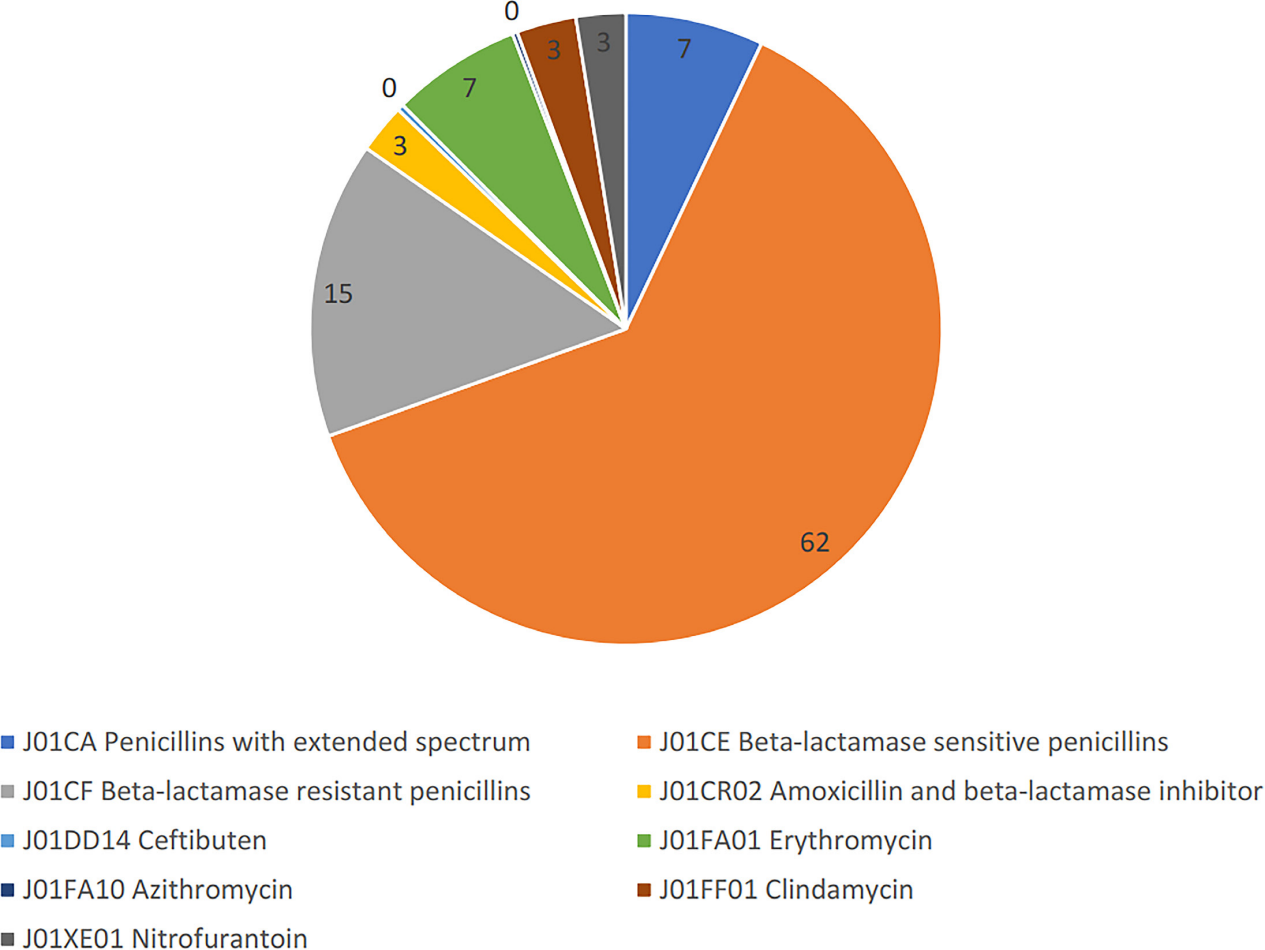
Distribution (percent of prescriptions) of selected antibiotics

This data on antibiotics prescriptions included 28 236 unique prescriptions over a 10-year period to a total number of 10 414 (61.1%) ABIS children. The other ABIS children (38.9%) received no antibiotic prescription during this period. Each prescription-receiving patient had a mean of 2.7 prescriptions. At the 5-year follow-up, 6231 (83.7%) of the 7443 participants had received and filled antibiotic prescriptions. Beta-lactamase sensitive penicillins (J01CE) was the dominant group, with 62% of prescriptions. The distribution of the different included antibiotics is illustrated in Figure 3.

In the univariate analysis, using the number of antibiotic prescriptions per child as the dependent variable, a significant correlation between household income and number of prescriptions for the lowest three income quintiles (Q5 having OR = 1) was found. Higher income meant lower prescription rate. Sex was significantly related (OR = 1.12, *P*<0.001) to antibiotic prescription; boys received more antibiotic prescriptions than girls (Table 2). The father’s educational level correlated significantly to the child’s number of antibiotic prescriptions, the mother’s educational level did not. Parent-reported infections that required antibiotics correlated significantly to the number of antibiotic prescriptions.

In the multiple regression analysis, a significant association between the number of prescriptions and the household income was found, where higher income meant lower prescription rate. However, this was not significant at all income quintiles. Perhaps more important, a significant association between the parent-reported number of infections in children aged 2–5 years and the number of antibiotic prescriptions in children aged 5–14 years was found, even when adjusted for mother’s and father’s educational levels, sex, and household income (OR range = 1.24 to 2.31, *P*<0.001) (Table 3).

## Discussion

### Summary

This 10-year follow-up of antibiotic prescriptions for a cohort of Swedish children confirmed that the number of parent-reported antibiotic-treated infections in early childhood may be reflected in higher rates of prescribed antibiotics to these children later in childhood. These results also show that children in high-income families received fewer antibiotic prescriptions than children in low-income families.

### Strengths and limitations

One key strength of this study is the prospective population-based design of the ABIS study, with a large sample size. Another strength is that this data partly comes from reliable automated official records, not dependent on self-reporting.

Parent-reported number of antibiotics-treated infections at age 2–5 years was used as the predictor, which makes these results particularly interesting, as not many studies seem to have done this. Using doctors’ diagnoses versus prescription of antibiotics might have induced a circularity bias. However, there are limitations to self-reports because the reliability of the parents' recollected memories are not known.

The follow-up period is 10 years, and it is known that Swedish children visit the doctor or primary healthcare centre most frequently in their early years.^3^ This should mean that they receive a large proportion of their antibiotics prescriptions during their first years. The SPDR started in 2005, which limits the available data. Also unknown is the proportion of filled prescriptions where the antibiotic was for some reason never consumed.

### Comparison with existing literature

High parent-reported number of antibiotics-treated infections is reflected in a higher antibiotic prescription rate. This is a logical and expected result, and confirms the parents’ reports. However, there is no robust explanation why children with many infections at age 2–5 years also receive more prescriptions during the following 10 years. Although ear, nose, and throat (ENT) infections are more common among children from lower socioeconomic groups,^21^ they diminish as children get older.^3^ Also, many ENT infections are viral, not requiring antibiotics. Therefore, this does not explain all of the extra prescriptions in later years. One explanation may be that certain individuals show more symptoms of infections, and therefore continue to receive proportionally more antibiotics during childhood (and perhaps adulthood). Another contributing mechanism might be a family model of handling disease, especially for infection-prone children.

A degree of bidirectionality may be present. The higher prescribing rate for children from lower socioeconomic groups could reflect higher morbidity, as other studies have shown.^4,7^ This may explain a proportion of antibiotic treatments, especially in their early years. This may affect the way the family handles morbidity, by changing the perception of healthcare consumption from an exception into something more common, or with a lower threshold to ask for or accept antibiotics. It may reflect a medicalised family model, where seeing the doctor is a solution used more often than in other families. No question in this database gives a direct answer to this, so further research is warranted.

Although relatively weak, some socioeconomic gradients in antibiotics prescriptions were found for this cohort. Melander *et al* found stronger correlations between antibiotic usage and socioeconomic factors.^9^ On a municipality level, this might reflect the local medical culture or preferences of the local doctors. The present study’s data were analysed on an individual level and covered a large geographical area, partly confirming previous findings.

Several studies have found different morbidity rates between socioeconomic groups,^4,22^ where children of families of lower socioeconomic backgrounds have more infections than children of families of higher socioeconomic backgrounds. This could be relevant for the present study cohort. If children of families of lower socioeconomic backgrounds had the same number of prescriptions, this suggests an underconsumption of drugs (and possibly other health benefits). But the present study shows that children of families of higher socioeconomic backgrounds receive fewer antibiotics, confirmed by their lower parent-reported infection rates.

This study hypothesised that highly educated parents might be sceptical towards antibiotics, perhaps having a wider array of information sources. Also hypothesised, low-income parents, due to income loss from taking care of a child at home, might ask for antibiotics to get the child back to school quicker. This study's results do not contradict these hypotheses, but the parents’ motives were not known. What was found was that belonging to the upper income quintile seems to have a slight protective effect from parent-recollected antibiotic-treated infections and antibiotic consumption, differing significantly from the lower three income quintiles.

Most prescription drugs in Sweden, including antibiotics, are discounted or free of charge to the patient after certain levels of co-payment have been achieved (a typical course of penicillin for a child costs 10–15 EUR, while seeing the doctor is free. For 2017, the upper limit of co-payment was 225 EUR, which equals 0.5% of the mean pre-tax income per year in Sweden). This means that children of familes of higher socioeconomic backgrounds have no large economic advantage over children of families of lower socioeconomic backgrounds in regard to treatment of health issues. Also, there are no economic incentives for physicians to treat their infections differently. Parents in Sweden can stay at home when their child is ill, with a benefit of approximately 80% of their normal wages^3^ up to a certain level. This means that high-income parents lose proportionally more money than low-income parents. The authors believe economic reasons have no large impact on antibiotic prescription and consumption.

The number of prescriptions per child appears low compared to many other countries.^20^ Furthermore, beta lactamase-sensitive penicillin as drug of choice may seem uncommon. The low antibiotic resistance in Sweden explains this in part. Swedish doctors are expected to comply with a number of regulations regarding the use of antibiotics, regardless of the patient’s socioeconomic status. All hospitals and county councils have regional drug guidelines. The Swedish national strategic programme against antibiotic resistance (Strama) advocates rational usage of antibiotics. The Strama guidelines^19^ are generally accepted among Swedish doctors. Kamps *et al* found that GPs seem to adhere to guidelines of a regional formulary.^24^ Of course, not all doctors will act in unison,^25,26^ and many other factors also influence doctors’ behaviour.^16,27–30^

Most health care for Swedish children is provided by public primary healthcare centres, and only a small fraction by private centres or practices. These private centres are mainly financed by county councils, use the common electronic patient records, and take part in the public antibiotics strategy. Common records and booking systems obstruct 'shopping around' for antibiotic prescriptions. The remuneration system does not encourage prescribing, so there is no direct financial gain for (unnecessary) prescription of antibiotics.

## Implications for practice

The study findings may either reflect that an increased morbidity persists for some individuals during a longer period during childhood, or reflect a continued pattern of higher healthcare utilisation, regardless of actual morbidity.

General guidelines of antibiotics prescription leave less room for personal medical judgements, which may explain in part why this study found only a small socioeconomic influence on prescription of antibiotics in Swedish primary health care. Nevertheless, continued awareness among GPs is important to ensure equal treatment according to the needs of all children, regardless of socioeconomic status.
